# Aristolochic acid I and ochratoxin A differentially regulate VEGF expression in porcine kidney epithelial cells—The involvement of SP-1 and HIFs transcription factors

**DOI:** 10.1016/j.toxlet.2011.04.022

**Published:** 2011-07-28

**Authors:** Anna Stachurska, Magdalena Kozakowska, Alicja Jozkowicz, Jozef Dulak, Agnieszka Loboda

**Affiliations:** Department of Medical Biotechnology, Faculty of Biochemistry, Biophysics and Biotechnology, Jagiellonian University, Gronostajowa 7, 30-387 Kraków, Poland

**Keywords:** AA, aristolochic acid, AAI, aristolochic acid I, AAII, aristolochic acid II, AA-ATN, aristolochic acid-induced acute tubular necrosis, AAN, aristolochic acid-induced nephropathy, AdGFP, adenoviral vectors containing GFP cDNA, AdHIF-1,-2α, adenoviral vectors containing HIF-1,-2α cDNA, β-gal, β-galactosidase, BEN, Balkan endemic nephropathy, CKDs, chronic kidney diseases, EMT, epithelial to mesenchymal cell transformation, GFP, green fluorescent protein, HIF, hypoxia inducible factor, HRE, hypoxia responsive element, HRP, horseradish peroxidase, LDH, lactate dehydrogenase, LLC-PK1, porcine kidney epithelial cell line, IARC, The International Agency for Research on Cancer, OTA, ochratoxin A, ROS, reactive oxygen species, RT, room temperature, TGFβ, transforming growth factor β, VEGF, vascular endothelial growth factor, Nephropathy, Kidney diseases, Vascular endothelial growth factor, Angiogenesis, Hypoxia, LLC-PK1

## Abstract

Aristolochic acid I (AAI) and ochratoxin A (OTA) cause chronic kidney diseases. Recently, the contribution of hypoxic injuries and angiogenic disturbances to nephropathies has been suggested, but underlying mechanisms have not been fully clarified yet.

In porcine kidney epithelial cell line, LLC-PK1 cells, treatment with non-toxic doses of AAI increased whereas with OTA decreased production of vascular endothelial growth factor (VEGF), the angiogenic factor with well-defined functions in kidney. Moreover, the activity of transcription factors regulating VEGF expression was differentially affected by examined compounds. Activity of hypoxia inducible factors (HIFs) and SP-1 was increased by AAI but diminished by OTA. Interestingly, AP-1 activity was inhibited while NFκB was not influenced by both toxins. Mithramycin A, a SP-1 inhibitor, as well as chetomin, an inhibitor of HIFs, reversed AAI-induced up-regulation of VEGF synthesis, indicating the importance of SP-1 and HIFs in this effect. Additionally, adenoviral overexpression of HIF-2α but not HIF-1α prevented OTA-diminished VEGF production suggesting the protective effect of this isoform towards the consequences exerted by OTA.

These observations provide new insight into complex impact of AAI and OTA on angiogenic gene regulation. Additionally, it adds to our understanding of hypoxia influence on nephropathies pathology.

## Highlights

► AAI increases whereas OTA decreases production of proangiogenic VEGF. ► The upregulation of VEGF expression by AAI is caused by induction of SP-1 and HIFs. ► Hypoxia prevents OTA-diminished VEGF production ► The effect of hypoxia on OTA-reduced VEGF is mediated by HIF-2α but not HIF-1α.

## 1 Introduction

Aristolochic acid (AA), a chemical found in *Aristolochia* and *Asarum* species, is present in a number of botanical products sold as “traditional medicines”, dietary supplements or weight-loss remedies. AA is a ∼1:1 mixture of two forms, aristolochic acid I (AAI) and aristolochic acid II (AAII), of which the first has higher nephrotoxicity in cellular and animal models (Shibutani et al., 2007). AA is a rodent carcinogen and was responsible for aristolochic acid-induced nephropathy (AAN) among women under slimming regime in Belgium and China (Arlt et al., 2002). Moreover, it is one of the possible causative agents of Balkan endemic nephropathy (BEN) (Stefanovic et al., 2006). The major targets of AA-induced toxicity are kidneys and urothelial tracts (Stiborova et al., 2008). AA was reported to be among the most potent 2% of known carcinogens and herbal remedies contaminated with *Aristolochia* were classified as carcinogenic to humans (Group 1) by the International Agency for Research on Cancer (IARC) (Arlt et al., 2002; IARC, 2002).

BEN development is also closely correlated with the occurrence of ochratoxin A (OTA), one of the mycotoxins produced by members of *Aspergillus* and *Penicillium* family (O’Brien and Dietrich, 2005; Pfohl-Leszkowicz and Manderville, 2007). The presence of this compound is proven for plant-derived products such as cereals, coffee and bread. Still, it was also detected in cocoa, nuts, dried vine fruits, grains as well as in wine. Pork and food products from pigs fed with contaminated grain may also be a source of OTA, what is linked to the high stability of OTA and its long half-life in blood and tissues (International Programme on Chemical Safety, 1990). The dose of OTA may vary in food from 0.5 mg/kg in baby foods to 10 mg/kg in soluble coffee and dried vine fruits (Coronel et al., 2010) and the tolerable intake was estimated by European Commission (1997) at 5 ng/kg body weight/day. The data from OTA presence in plasma indicated the geographical differences, being the lowest in Japanese people and the highest in Argentina. The assessed level of OTA in plasma in healthy people was 0.15 (min), 0.45 (mean) and 9.15 (max) ng OTA/ml plasma (Coronel et al., 2010). Significantly higher blood serum or plasma levels of OTA in patients with kidney or urinary disorders compared to healthy people have been reported, with an exceptionally high level of 1800 ng/ml found in one of the serum Croatian samples (reviewed in Scott, 2005). OTA, similarly to AA, is toxic mainly for kidney in domestic and laboratory animals, and it was classified by IARC as a possible human carcinogen (group 2b) (IARC, 1993). Some data showed, however, a tendency in the direction of group 2A toxicity (reviewed in Kuiper-Goodman, 1996).

Recently, apart from well-established features of AAN and BEN including tubular proteinuria, the progressive fibrosis, the epithelial to mesenchymal cell transformation (EMT), proximal tubule apoptosis and kidney size reduction (Vukelic et al., 1992; Yang et al., 2007), the changes in the kidney vasculature have been suggested. In BEN the microvascular hyalinosis/sclerosis were found (Ferluga et al., 1991), whereas in AAN the impairment of vascular network is connected with existence of severe hypoxia caused by the reduction of peritubular capillary density (Sun et al., 2006a).

Hypoxia inducible factors (HIFs) are transcription factors stabilized under hypoxic conditions, what leads to their nuclear translocation and further to induction of various genes, like pro-angiogenic vascular endothelial growth factor (VEGF) (Zagorska and Dulak, 2004). VEGF plays a crucial role in kidney, where it is produced mostly by glomerular epithelial cells (podocytes) but was also found in epithelial cells of the collecting and distal tubules as well as in nephron's proximal tubules (Baderca et al., 2006). It is responsible for the maintenance of the fenestrated phenotype of glomerular epithelial cells as well as it facilitates the high rate of glomerular ultrafiltration (Maharaj and D’Amore, 2007). Moreover, the perturbances in its expression in tubular cells was found in different kidney diseases, like in diabetic nephropathy (Lindenmeyer et al., 2007) and progressive proteinuric renal failure (Rudnicki et al., 2009). In patients with chronic kidney diseases (CKDs) (Futrakul et al., 2008) and with the chronic allograft nephropathy (Hotchkiss et al., 2006) expression of VEGF is strongly down-regulated. In addition to HIFs, multiple transcription factors, like SP-1, AP-1 or NFκB, are known to regulate the expression of VEGF (Pages and Pouyssegur, 2005). SP-1, which is involved in many cellular processes, such as cell cycle regulation, differentiation and angiogenesis, is also connected with fibrosis by affecting transforming growth factor-β (TGFβ) pathway (Kum et al., 2007; Sysa et al., 2009). Therefore, SP-1 activity may be important in the AA and OTA-induced toxicity.

Although there are evidences indicating that changes in vasculature may be one of the mechanisms observed in kidney diseases, systematic studies on the role of toxins in VEGF regulation as well as the involvement of hypoxia/ischemia in the pathogenesis of different renal diseases are missing. Therefore, the objective of the present study was to compare AAI and OTA impact on VEGF expression as well transcription factors regulating its expression in cultured kidney tubulus cells. Comparison between effects of both toxins on VEGF expression may add to our understanding of the role of these toxins in nephropathy development.

## 2 Materials and methods

### 2.1 Chemicals

Aristolochic acid I (AAI), ochratoxin A (OTA), mithramycin A, thiazolyl blue tetrazolium bromide (MTT), dichlorofluorescein diacetate (DCFH-DA) and SYBR Green were obtained from Sigma–Aldrich. Oligo(dT) primers, dNTP's, M-MLV reverse transcriptase, Non-radioactive Cytotoxic Lactate Dehydrogenase (LDH) assay and Luciferase Activity Assay were obtained from Promega and chetomin was from Alexis Biochemicals. The ELISA kit for human VEGF was procured from R&D Systems Europe. The cell proliferation BrdU ELISA kit was bought from Roche, the Great Escape SEAP Chemiluminescent Detection kit was from Clontech BD Biosciences and SuperFect Transfection Reagent was procured from Qiagen. High glucose DMEM medium was from Cytogen. Fetal bovine serum (FBS) and antibiotics (penicillin, streptomycin) were from PAA. Rabbit polyclonal anti-HIF-1α (cat no. sc-10790) and anti-HIF-2α (cat no. sc-28706) antibodies were purchased from Santa Cruz Biotechnology, mouse monoclonal anti-α-tubulin (cat no. CP06) was from Calbiochem, anti-rabbit IgG conjugated with horseradish peroxidase (HRP) (cat no. 7074) was from Cell Signaling Technology whereas anti-mouse IgG conjugated with HRP (cat no. 32230) was from Pierce. Goat anti-rabbit IgG conjugated with Alexa Fluor 568 (cat no. A21069) was from Invitrogen. Mounting medium with DAPI was bought from Vector Laboratories.

### 2.2 Cell culture and incubation experiments

LLC-PK1 cell line, an established epithelial cell line derived from porcine renal cortex, was kindly supplied by Prof. Gerald Rimbach (Institute of Human Nutrition and Food Science, Christian Albrechts University Kiel, Germany). The cells were passed in high glucose DMEM medium, supplemented with 10% FBS, streptomycin (100 U/ml) and penicillin (100 g/ml), and kept under standard conditions (37 °C, 5% CO_2_). Toxins were prepared as a 50 mM stock solution (AAI in DMSO, and OTA in methanol). In experiments with mithramycin A and chetomin, cells were pretreated for 30 min with mithramycin A or with chetomin, and then costimulated with AAI for next 24 h. For hypoxia experiments, cells were treated with OTA and then put into hypoxic conditions (0.5% O_2_, 5% CO_2_, 94% N_2_) for 24 h.

### 2.3 Cytotoxic tests

The effect of AAI (1–100 μM) and OTA (2.5–100 μM) on porcine kidney cell viability has been determined by non-radioactive cytotoxic LDH assay and MTT conversion according to provider's instruction.

### 2.4 Cell proliferation

LLC-PK1 cells were seeded at a density of 5,000 cells per well in a 96-well plate.

After 24 h non-confluent cells were stimulated by OTA and AAI and then cell proliferation was assessed by bromodeoxyuridine incorporation by the use of BrdU ELISA kit according to manufacturer's instructions.

### 2.5 Transfection with reporter plasmids

LLC-PK1 cells growing to 70% confluence in 24-well plates were transfected as described previously (Loboda et al., 2005) with construct containing full VEGF promoter or hypoxia responsive element (HRE) fragment of VEGF promoter (kindly provided by Dr. Hideo Kimura, Chiba, Japan). The pAP-1-SEAP and pNFκB-SEAP vectors, containing the AP-1 and NFκB binding regions, respectively, connected to secreted alkaline phosphatase (SEAP) reporter gene were purchased from Clontech. The SP-1-luc plasmid, containing the upstream region of the VEGF promoter from −135 to +3 bp, cloned into pAH1409 vector was kindly delivered by Dr Ulrike Fiedler (Tumor Cell Biology, Freiburg, Germany). The pCMV-lacZ plasmid containing the β-galactosidase (β-gal) gene driven by CMV promoter was from Promega and was co-transfected to cells together with one of the above described reporter vectors. The activity of reporter gene, luciferase, β-gal or SEAP was determined in cell lysates or cell culture media, respectively. Determination of luciferase enzyme activity was done according to manufacturer's protocol using Tecan plate reader. Chemiluminescent SEAP assay was performed according to the vendor's protocol with a modification, as described previously (Boesch-Saadatmandi et al., 2008).

### 2.6 Transduction of the cells with adenoviral vectors

Adenoviral vectors containing HIF-1α or HIF-2α cDNA (AdHIF-1α, AdHIF-2α) were a kind gift from Prof. Seppo Yla-Herttuala (Kuopio, Finland) and Prof. Lorenz Poellinger (Stockholm, Sweden). The pAdHIF-1α was generated as described previously (Pajusola et al., 2005). Briefly, construct was stabilized against prolyl hydroxylation and subsequent ubiquitin-mediated proteolytic degradation in normoxic conditions by point mutations (P402A/P563A). A control vector (AdGFP) was produced using the Adeno-X system as described previously (Loboda et al., 2009).

### 2.7 Reverse transcription–polymerase chain reaction and real-time PCR

RNA isolation and RT-PCR were performed as described previously (Loboda et al., 2005). Quantitative RT-PCR was performed using StepOnePlus™ Real-Time PCR Systems (Applied Biosystems). The real-time PCR reaction mixture, equalized with ultra pure water to 15 μl, contained 7.5 μl of SYBR Green, 0.75 μl of both reverse and forward primer, and 50 ng of cDNA. Specific primers for VEGF (5′ CTG GTC TTG GGT GCA TTG 3′; 5′ CAC CGC CTC GGC TTG TCA CAT 3′), HIF-1α (5′ TGC TTG GTG CTG ATT TGT GA 3′; 5′ GGT CAG ATG ATC AGA GTC CA 3′), HIF-2α (5′ TCC GAG CAG TGG AGT CAT TCA G 3′; 5′ GTC CAA ATG TGC CGT GTG AAA G 3′), SP-1 (5′ AAG AAG GGA GGC CCA GGT GTA G 3′; 5′ CAT GAC GTT GAT GCC ACT GTT G 3′) and constitutive EF2 (5′ GCG GTC AGC ACA ATG GCA TA 3′; 5′ GAC ATC ACC AAG GGT GTG CAG 3′) have been used.

### 2.8 ELISA assays

Cell culture media were collected and concentration of VEGF protein was quantified following the manufacturer's protocol.

### 2.9 Immunocytochemistry

Cells were seeded on eight-chamber culture slides (BD-Falcon). After 24 h of stimulation with AAI and OTA, cells were fixed (20 min, 4% formaldehyde, RT), washed three times with PBS and permeabilized (20 min, 0.1% Triton X100 in PBS, RT). After washing three times with PBS and blocking (1 h, 3% BSA in PBS, RT), primary rabbit IgG antibodies: anti-HIF-1α and anti-HIF-2α antibodies, respectively, were added (overnight, 1:300 in 3% BSA in PBS, 4 °C). After washing three times for 5 min with PBS, incubation with the secondary anti-rabbit IgG antibody conjugated with AlexaFluor (30 min, 1:1000 in 3% BSA in PBS, RT) and washing three times for 5 min with PBS, mounting medium with DAPI was used. The observation of specimens was done by the use of fluorescent microscope with green and UVA filter in order to detect red fluorescence and blue signal from AlexaFluor and DAPI, respectively. In negative control chambers the primary antibodies were omitted in order to verify the level of autofluorescence and unspecific binding.

### 2.10 Western blotting

Total cellular protein was isolated from LLC-PK1 cells and western blotting was performed as described previously (Loboda et al., 2005). Rabbit polyclonal anti-HIF2α (Santa Cruz Biotechnology, cat no. sc-28706) and mouse monoclonal anti-α-tubulin (Calbiochem, cat no. CP06) antibodies were used followed by incubation with the secondary antibodies (anti-rabbit HRP–Cell Signaling, cat no. 7074 and anti-mouse HRP–BD Biosciences cat no. 554002, respectively) and Super Signal WestPico Chemiluminescence Substrate.

### 2.11 Determination of intracellular reactive oxygen species generation using DCFH-DA oxidation

The assay was performed by the use of DCFH-DA (10 μM) which was added for the last hour of incubation. The fluorescence (excitation 485 nm, emission 535 nm) was measured from cell lysates. Obtained data were normalized to protein concentration values. As a positive control for test 4 h stimulation with PGJ2 was used.

### 2.12 Statistical analysis

All experiments were performed in duplicates and were repeated at least three times unless otherwise indicated. Data are presented as mean ± SD. Statistical evaluation was done by analysis of variance (ANOVA), followed by a Bonferoni post hoc test for multiple comparisons, or with Student's *t*-test for two group comparisons. Differences were accepted as statistically significant at *p* < 0.05.

## 3 Results

### 3.1 AAI and OTA attenuate LLC-PK1 proliferation

**Fig. 1:**
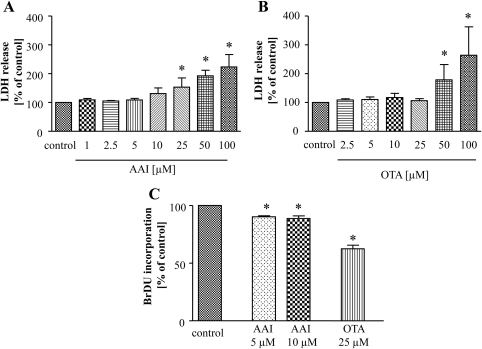
AAI and OTA affect LLC-PK1 cell viability and proliferation. Cells were cultured in the presence of increasing concentrations of AAI and OTA for 24 h. Cell viability, assessed by measurement of LDH release showed that 10 μM AAI (A) and 25 μM OTA (B) are the highest non-toxic concentrations. After 24 h stimulation with non-toxic concentration of both toxins analysis of BrdU incorporation pointed out inhibition of cell proliferation after delivery of 5 and 10 μM AAI as well as after 25 μM OTA (C). Mean of at least 3 experiments performed in duplicates; *p < 0.05 vs control

Firstly, we determined the effect of AAI (1–100 μM) and OTA (2.5–100 μM) on the viability of porcine kidney LLC-PK1 cells. Using the LDH release assay we found that the highest non-cytotoxic concentration of AAI was 10 μM and of OTA was 25 μM (Fig. 1A and B). As the results of MTT test (data not shown) were in accordance to LDH assay such doses were chosen as the maximal ones for all further experiments.

Then we measured cells proliferation and we showed that AAI as well as OTA at non-toxic doses inhibited BrdU incorporation and caused attenuation of LLC-PK1 proliferation (Fig. 1C).

### 3.2 AAI and OTA differentially regulate VEGF production

**Fig. 2:**
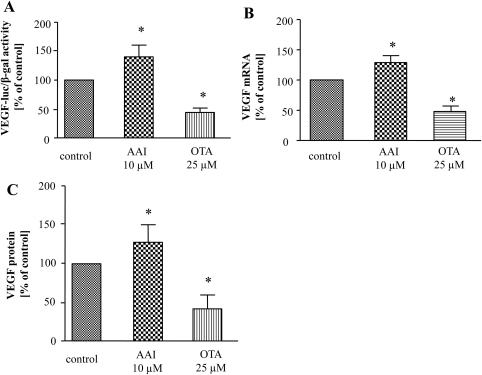
AAI elevates whereas OTA diminishes VEGF production in LLC-PK1 cells. LLC-PK1 cells were transfected with reporter plasmid containing luciferase encoding sequence under the control of VEGF promoter and then stimulated with toxins. The elevation of VEGF promoter activity 24 h after AAI and diminishment after OTA delivery was observed (A). Analysis of mRNA level by real-time PCR (B) and protein production by ELISA (C) indicated the enhancement and down-regulation of VEGF expression after AAI and OTA adding, respectively. Mean of 3 experiments performed in duplicates; *p < 0.05 vs control.

We investigated the effect of both toxins on expression of VEGF, main pro-angiogenic factor with well-defined functions in kidney (Maharaj and D’Amore, 2007). VEGF transcription was activated by AAI as determined by luciferase assay in cells transfected with a reporter plasmid containing a human full-length VEGF promoter (Fig. 2A) as well as evidenced by increased VEGF mRNA expression (Fig. 2B). In contrast, the effect of OTA on VEGF transcription was inhibitory, both on the VEGF promoter activity (Fig. 2A) and mRNA level (Fig. 2B). Basal VEGF protein production in LLC-PK1 cells ranges around 200–300 pg/ml and it was influenced by both toxins comparable to mRNA level. ELISA test demonstrated that AAI slightly but significantly elevated, whereas OTA strongly decreased VEGF protein level (Fig. 2C).

### 3.3 AAI and OTA influence activity of transcription factors

In order to investigate the potential mechanisms of alterations in VEGF production we checked the effect of AAI and OTA on the activity of transcription factors known to regulate VEGF expression (the binding sites of which are located within VEGF promoter), such as HIFs, SP-1, AP-1 and NFκB (Pages and Pouyssegur, 2005).

**Fig. 3:**
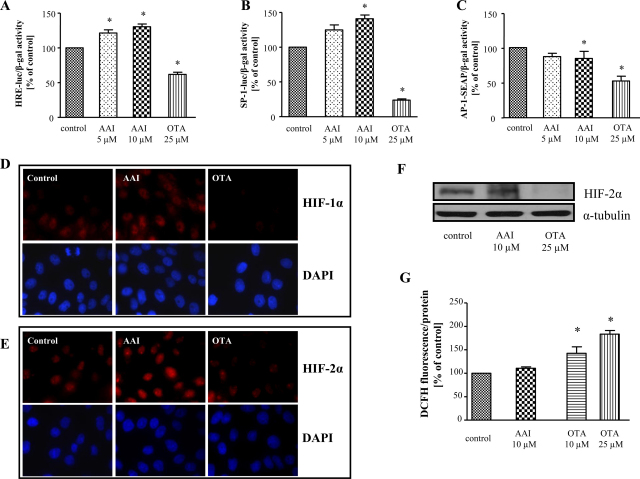
AAI and OTA differentially regulate the activity of HRE and SP-1 but both attenuate AP-1 activity. 24 h after transfection with HRE-luc (A), SP-1-luc (B) and AP-1-SEAP (C) reporter plasmids LLC-PK1 cells were stimulated with AAI and OTA for next 24 h. Analysis demonstrated AAI-elevated and OTA-diminished HRE activity (A). SP-1 activity was enhanced after AAI and down-regulated after OTA (B) adding. Both AAI and OTA diminished AP-1 activity (C). Immunocytochemical staining showed that the stabilization of HIF-1α (D) and HIF-2α (E) was elevated after AAI (middle column) but decreased after OTA (right column) delivery. HIF-2α expression was increased by AAI and diminished by OTA as shown by Western blot (F). ROS generation was increased after OTA but not after AAI stimulation (G). Mean of 3 (A, B, G) and 2–3 (C) experiments performed in duplicates, D, E-representative immunofluorescent staining, F–representative western blot; *p < 0.05 vs control.

Using the cells transfected with a reporter construct regulated by the hypoxia responsive element (HRE) from the VEGF promoter we demonstrated that AAI activated whereas OTA diminished HRE activity (Fig. 3A) at concentrations tested. Moreover, we showed that AAI and OTA exerted opposite effect on SP-1 activity (Fig. 3B). AAI was found to produce increase in SP-1 activity (Fig. 3B) but it did not affect SP-1 mRNA level (Fig. S1A). In contrast, OTA reduced activity of SP-1 (Fig. 3B) and SP-1 mRNA level was concomitantly inhibited by ∼42 ± 18%. Additionally, AP1-SEAP construct was employed to determine the effect of toxins on AP-1 activity. As observed previously (Boesch-Saadatmandi et al., 2008) and confirmed in this study, OTA diminished AP-1 activity. AAI delivery exerted also inhibitory effect (Fig. 3C), although not so strong as OTA. In our hands, the activity of NFκB transcription factor was not influenced by non-toxic doses of AAI and OTA (Fig. S1B).

In order to verify the effect of both toxins on HIFs transcription factors activity we have performed the immunofluorescent staining as well as western blot for specific HIF isoforms. Stimulation with AAI elevated nuclear accumulation of HIF-1α and HIF-2α isoforms (Fig. 3D, E, middle column) whereas after OTA delivery inhibition was observed (Fig. 3D, E right column). Also western blot analysis of HIF-2α protein revealed inhibition after OTA and up-regulation caused by AAI stimulation (Fig. 3F). As ROS are known to affect HIF level (reviewed in Stachurska et al., 2010) in order to verify the possible mechanism of alterations in HIF level we investigated the effect of AAI and OTA on ROS generation. We observed previously (Boesch-Saadatmandi et al., 2008) as well as in this study, the enhancement of ROS generation after OTA delivery, however AAI did not affect ROS level (Fig. 3G). Therefore, increase in HIFs evoked by AAI is not caused by ROS.

### 3.4 AAI increases VEGF expression via SP-1 and HIF transcription factors

**Fig. 4:**
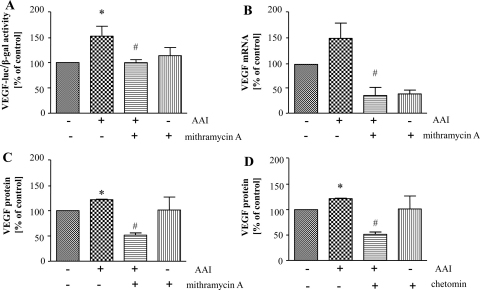
Induction of VEGF by AAI goes through SP-1 and HIFs activation. LLC-PK1 cells were transfected with reporter plasmid containing luciferase encoding sequence under the control of VEGF promoter. 24 h after transfection cells were prestimulated with 1 μM mithramycin A (A–C) or 60 nM chetomin (D) for 30 min and then AAI was added for 24 h. The elevation of VEGF promoter activity (A), mRNA level (B) as well as VEGF protein level (C) evoked by AAI was reversed by inhibition of SP-1 activity by mithramycin A. Moreover, inhibition of HIF activity by chetomin caused the reversal effect on AAI-induced VEGF protein level (D). Mean of 3 (A, B, C) or 2 (D) experiments performed in duplicates; *p < 0.05 vs control; ^#^*p* < 0.05 vs AAI-treated cells.

As AAI concomitantly elevates VEGF expression and activity of SP-1 and HIFs, we investigated the possible role of SP-1 and HIFs transcription factors in induction of VEGF production evoked by AAI. Mithramycin A was used to silence SP-1 activity (Blume et al., 1991) whereas HIFs were inhibited with chetomin (Kung et al., 2004). We found out that AAI-induced VEGF transcription, both promoter activity (Fig. 4A) and mRNA level (Fig. 4B) were attenuated by 1 μM mithramycin A. Similar effect was also observed on VEGF protein level (Fig. 4C). In addition, 60 nM chetomin attenuated AAI-induced VEGF protein production measured by ELISA (Fig. 4D) suggesting also the role for HIFs in observed effect. However, AAI did not affect hypoxia-enhanced HRE activity (Fig. S2A) and hypoxia-induced VEGF production (Fig. S2B).

### 3.5 Hypoxia and HIF-2α but not HIF-1α attenuate the effect of OTA on VEGF production

**Fig. 5:**
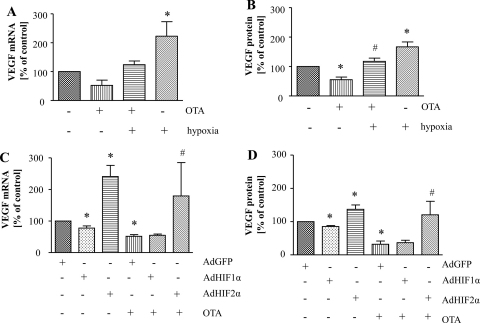
Hypoxia and HIF-2α attenuate the diminishment of VEGF production evoked by OTA. LLC-PK1 cells were cultured under normoxic and hypoxic conditions for 24 h and stimulated by 25 μM OTA. OTA attenuated induction of VEGF expression evoked by hypoxia on mRNA level determined by real-time PCR (A) as well as on the protein level assessed by ELISA (B). Overexpression of HIF-2α but not HIF-1α abolished the diminishment of VEGF expression evoked by OTA on mRNA (C) and protein (D) level. Mean from 2 (A), 3 (B), 3–5 (C) or 3–4 (D) experiments performed in duplicates, *p < 0.05 vs. control (A, B); vs. relevant AdGFP control (C, D), ^#^*p* < 0.05 vs. OTA (A, B); vs. AdGFP + OTA (C, D).

In order to investigate the possible involvement of HIFs in the observed down-regulation of VEGF by OTA in LLC-PK1 cells, firstly we verified the effect of OTA stimulation in hypoxic conditions. Basal level of VEGF was induced after 24 h of culturing of cells in 0.5% O_2_ and decrease of VEGF production caused by OTA was reversed by hypoxia (Fig. 5A, B). We also investigated the effect of OTA and hypoxia on HRE activity and we found that OTA diminished hypoxia-enhanced HRE activity (data not shown).

As both HIF-1 and HIF-2 transcription factors may mediate the hypoxic response, we investigated which HIF isoform is involved in the decrease of VEGF by OTA. For this purpose we used adenoviral vectors harboring encoding sequences of stable HIF-1α or HIF-2α, which allowed for significant increase in the expression of both isoforms with any mortality (data not shown). Adenoviral overexpression of HIF-2α but not HIF-1α caused increase of basal VEGF level as well was able to reverse the diminishment of VEGF production by OTA, suggesting that HIF-2 is crucial for the observed effects in kidney tubular cells (Fig. 5C, D).

## 4 Discussion

The carcinogenic effects of aristolochic acid (AA) and ochratoxin A (OTA) are widely described. Despite many trials aiming to discover the mechanism of their involvement to nephropathy progression, the sequence of events is still not clear.

The two main components of AA, AAI and AAII are responsible for nephropathy progression, however AAI is more potent cytotoxic agent towards kidney epithelium (Arlt et al., 2002; Liu et al., 2009). Nephrotoxic activity of OTA is well-documented, however, species-dependent discrepancies between man, pig and rodents are underlined. Such variations may be caused by the differences in the binding of OTA to serum proteins, oral bioavailability, the half-life of OTA in serum as well as in the different plasma clearance between species (reviewed in Petzinger and Ziegler, 2000). In the present study, porcine renal proximal tubule epithelial cells (LLC-PK1), a well characterized cell line often used in toxicological studies (Dietrich et al., 2001) was chosen as a model for investigation. Importantly, the high susceptibility of pigs towards OTA and their importance for livestock production is well-known and pork as well as food products from pigs fed with contaminated grain may also be a source of OTA (International Programme on Chemical Safety, 1990). Moreover, it is well established that proximal tubular cells are the main site of toxicity of both OTA and AAI. Proximal tubule injury is observed in aristolochic acid nephropathy in rats (Mengs, 1987; Lebeau et al., 2005) and analysis of both kidney functions and renal biopsies from AAN patients showed increased tubular proteinuria, impairment of proximal tubule functions and tubular necrosis (Depierreux et al., 1994). OTA was shown to be removed by tubular, but not glomerular filtration to the urine and *in vivo* studies underlined that OTA affects the proximal part of the nephron (Groves et al., 1998).

In AAN (Depierreux et al., 1994; Yang et al., 2005) and other kidney diseases (Neusser et al., 2010) tubulointerstitial damage observed during kidney fibrosis may be the effect of blood vessel injury. In the proper vessel functioning an important role plays vascular endothelial growth factor (VEGF), which in kidneys is expressed both in podocytes and additionally in proximal tubular cells (Baderca et al., 2006), which are the main site of AAI as well as OTA injury. Moreover, both tubular and glomerular VEGF may play an important role in the maintenance of peritubular or glomerular capillaries. Diminished VEGF production may lead to decreased endothelial survival and angiogenesis as well as tubular damage by ischemia (reviewed in: Schrijvers et al., 2004). The importance of the alterations in VEGF expression in epithelial cells of proximal and distal tubules was shown in human diabetic nephropathy patients (Lindenmeyer et al., 2007) as well as in patients with progressive proteinuric renal failure (Rudnicki et al., 2009).

We investigated the effect of AAI and OTA on VEGF, the potent pro-angiogenic factor, which is claimed to affect the nephropathy progression. The data concerning the role of VEGF in development of AAN are still not clear, although it seems that regulation of VEGF expression plays an important role in this disease. VEGF expression was reported to be down-regulated in rats with chronic AAN (Sun et al., 2006b) as well as in acute AAN rat model (Wen et al., 2008). In contrast, it was shown that in AA-induced acute tubular necrosis (AA-ATN) VEGF expression is elevated in renal tubules compared to control group, nevertheless, the expression was lower than in antibiotic-induced ATN (Yang et al., 2007). In our study we observed the elevation of VEGF transcription as well as protein expression after AAI treatment in LLC-PK1 cells. Interestingly, we showed that OTA has different effect on VEGF production compared to AAI in short-term treatment as potent inhibition of VEGF expression in LLC-PK1 cells was observed after OTA stimulation. In male F344/N rats treated with OTA no alterations in urinary level of VEGF was found (Hoffmann et al., 2010), however, the level of VEGF in urine may differ from ones present in organs or in serum. As VEGF may be produced by podocytes as well as epithelial tubular cells in order to better understand the effects evoked by AAI/OTA it is important to determine the production of VEGF by both types of cells in *in vivo* studies.

In order to investigate the mechanism of differential effect of AAI and OTA on VEGF production we verified the effect of both toxins on the activity of transcription factors, which binding sites are present in VEGF promoter, such as HIFs, AP-1, NFκB or SP-1 (Pages and Pouyssegur, 2005). Our data indicate that both toxins exert complex effect on various transcription factors, and as the result they may differentially regulate VEGF expression. AAI treatment caused SP-1 and HIFs up-regulation, whereas AP-1 was inhibited after 24 h of toxin delivery. Similarly, OTA treatment diminished AP-1 activity, but it also potently down-regulated SP-1 and HIFs activity. Moreover, the activity of NFκB was influenced neither by AAI nor by OTA. Such complicated data show that, although both toxins induced kidney damage, the mechanisms are different and should be carefully investigated in details. Additionally, the effect may be cell-type dependent as in human HKC-8 cells only the effect of OTA on HRE and AP-1 activity was the same as in LLC-PK1 cells, whereas NFKB was induced and SP-1 activity was not affected by this toxin (Fig. S3). In pheochromocytoma PC-12 cells the inhibition of AP-1 (Oh et al., 2004), whereas in 12-day rat embryo midbrain cells the activation of this factor by OTA was observed (Hong et al., 2002). In contrast to our data, where we did not observe the alterations in NFκB activity after OTA delivery, such activation was shown in proximal OK cells (Sauvant et al., 2005), in immortalized human kidney epithelial cells (IHKE) (Rached et al., 2006) as well as in 12-day rat embryo midbrain cells (Hong et al., 2002). On the other hand, in LPS-activated RAW264.7 macrophages DNA binding activity of NFκB was considerably lower after AAI treatment in comparison to control cells (Liu et al., 2011). However, such differences may be caused by the dose and time of stimulation in individual experiment. In case of SP-1, there are no data concerning the effect of OTA on activity of this transcription factor, so we have shown for the first time the diminishment of SP-1 activity after OTA. Moreover, our results indicating inhibitory effect of OTA on HRE activity and HIFs transcription factors are also unique. To our best knowledge, only one paper showing increased mRNA level for HIF-1α in rat proximal tubule cells after OTA treatment was published already but the protein level was not investigated (Luhe et al., 2003). However, in case of HIF proteins, the protein stability is much more crucial, therefore these data and our data do not exclude each other.

The knowledge about AA influence on the activity on transcription factors is also very limited. We have presented for the first time that AAI exerts opposite effect than OTA on SP-1 and AP-1, enhancing and diminishing their activity, respectively. The already published data about the effect of AA on NFκB is contradictory, as inhibition in human HK-2 cells (Chen et al., 2010) and induction in kidney of Hupki (human TP53 knock-in) mice (Arlt et al., 2011) has been observed. Interestingly, in our hands activity of NFκB was not affected but we observed HIF induction after AAI delivery. These data are in accordance with results from animal studies. The presence of hypoxia was also observed in male Wistar rats treated with AAI for 4 days (Cao et al., 2010). In rat model AA evoked elevated nuclear staining for HIF-1α with concomitant reduction in VEGF production in long (8–16 weeks) (Sun et al., 2006a,b) and short (4–7 days) term (Wen et al., 2008) experiments. Moreover, this increase of nuclear HIF-1α was present in the tubular cells in damaged area (Wen et al., 2008). However, in our studies concomitantly with HIF stabilization we observed elevation of VEGF production. The discrepancies between our results and published data may come from different time of stimulation and species-dependent differences in response. Additionally, it is possible that in case of longer AA treatment other transcription factors known to regulate VEGF expression, like AP-1, may play a role. Therefore, it seems that regulation of VEGF expression after delivery of AAI is much more complex. Thus, the understanding of the sequence of events evoked by AA is important to identify the origin of AAN development and still needs to be clarified.

The most important part of our study is the discovering of the possible mechanism of AAI/OTA action on VEGF production. The augmentation of HIFs and SP-1 transcription factors activity by AAI was paralleled with the up-regulation of VEGF transcription and protein level. By the use of mithramycin A, an inhibitor of SP-1 activity, and chetomin, an inhibitor of HIFs, we showed that AAI-elevated VEGF production is reversed after inhibition of SP-1 and HIFs, what confirms the role of these transcription factors in the effect of AAI on VEGF expression.

The next salient finding of our study is that hypoxia attenuated the inhibitory effect of OTA on VEGF production. In the kidney the localization of HIF isoforms depends on cell type with HIF-1α presence in the tubular epithelia, whereas HIF-2α expression mostly in endothelial, glomerular and interstitial cells (Rosenberger et al., 2005). Although different role of HIF isoforms in kidney development may be the result of divergent localization in cells, it is well documented that HIF-1 and HIF-2 also differs in regulation of gene expression (reviewed in Loboda et al., 2010). HIF stabilization elevates angiogenesis and therefore it may attenuate adverse effects of toxins delivery. On the other hand, HIF triggers also the expression of connective tissue growth factor (CTGF), which exhibit profibrotic effects (Higgins et al., 2004). Thus, long-term activation of HIF may lead to fibrosis development. Therefore the proper balance in HIF activation is crucial for therapeutic effect.

Our data indicate that in prevention of diminishment of VEGF production evoked by OTA in kidney proximal tubular epithelial cells HIF-2α but not HIF-1α plays a crucial role. Additionally, we found that HIF-1α overexpression diminished VEGF production, whereas only AdHIF-2α transduction resulted in elevation of VEGF expression. Therefore, it seems that two isoforms of HIF may play a distinct role in regulation of VEGF production in porcine proximal tubular epithelial cells, which are the major target of OTA action. Moreover, only HIF-2 exerts protective effect, especially against short-term acute kidney injuries. These results are in accordance with studies showing that HIF may be protective in acute renal injuries whether in case of chronic ones they exert opposite effect (Manotham et al., 2004). Still, the role of each HIF isoform in different kidney cell types may be various. Additionally, also the other factors, such as AP-1 and SP-1, should be investigated in this context.

In conclusion, we have shown complicated pattern of VEGF regulation by different toxins affecting kidney biology. To our knowledge, the influence of AAI and OTA on some transcription factors have not been investigated before and further investigations are necessary to analyze this intriguing effects.

## Conflict of interest

The author declares that there are no conflicts of interest.
