# Supplementary material for: Aristolochic acid I and ochratoxin A differentially regulate VEGF expression in porcine kidney epithelial cells—The involvement of SP-1 and HIFs transcription factors
Source: Toxicol Lett. 2011 Jul 28;204(2-3):118–26. doi: 10.1016/j.toxlet.2011.04.022 (PMC3154282; doi:10.1016/j.toxlet.2011.04.022)
Supplement: Supplementary file 1 [file mmc1.doc]

**Supplementary Figures**

**Fig.1. OTA diminished SP-1 mRNA level but neither OTA nor AAI influenced NFκB activity in LLC-PK-1 cells**

24h after stimulation with 25 μM OTA and 10 μM AAI RNA was isolated and the SP-1 expression on mRNA level was analyzed by the use of real-time PCR (A). Analysis demonstrated the diminishment of SP-1 mRNA level after OTA delivery. After transfection with NFκB-SEAP reporter plasmid (B) LLC-PK1 cells were stimulated with AAI and OTA for next 24h. Both toxins did not affect NFκB activity. Mean of 3 experiments performed in duplicates. * p<0.05 vs control.

**Fig.2. AAI did not affect hypoxia-enhanced HRE activity and hypoxia-induced VEGF production**

24h after transfection with HRE-luc (A) reporter plasmid LLC-PK1 cells were stimulated with AAI and were cultured under normoxic and hypoxic conditions for 24h. Induction of HRE activity by hypoxia was not influenced by AAI. Similarly, VEGF protein level in hypoxic conditions assessed by ELISA was not affected by AAI (B). Mean of 3 experiments performed in duplicates. * p<0.05 vs control.

**Fig. 3. In human HKC-8 cells OTA inhibited HRE and AP-1 activity with concomitant induction of NFκB.**

24h after transfection with HRE-luc, AP-1-SEAP, NFκB-SEAP and SP-1-luc reporter plasmids HKC-8 cells were stimulated with OTA for next 24h. Analysis demonstrated OTA-diminished HRE and AP-1 activity whereas NFκB activity was enhanced and SP-1 activity was not influenced by this toxin. Mean of 3 experiments performed in duplicates. * p<0.05 vs control.
