# Supplementary material for: Aristolochic acid I and ochratoxin A differentially regulate VEGF expression in porcine kidney epithelial cells—The involvement of SP-1 and HIFs transcription factors
Source: Toxicol Lett. 2011 Jul 28;204(2-3):118–26. doi: 10.1016/j.toxlet.2011.04.022 (PMC3154282; doi:10.1016/j.toxlet.2011.04.022)
Supplement: Supplementary file 2 [file mmc2.ppt]

## Slide 1
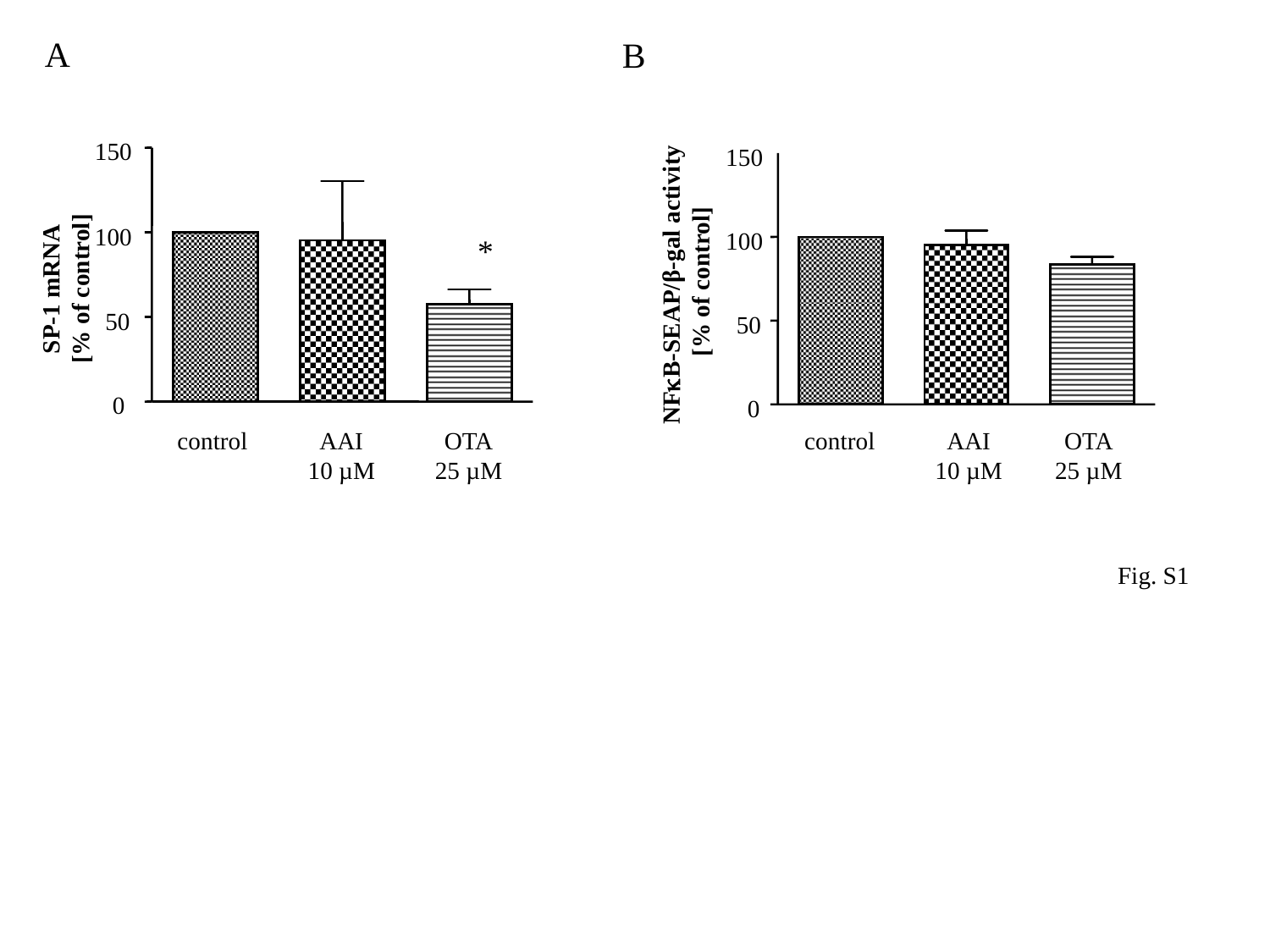

A
B
150
150
100
*
100
NFκB-SEAP/β-gal activity
 [% of control]
SP-1 mRNA
[% of control]
50
50
0
0
control
AAI
10 µM
OTA
25 µM
control
AAI
10 µM
OTA
25 µM
Fig. S1

## Slide 2
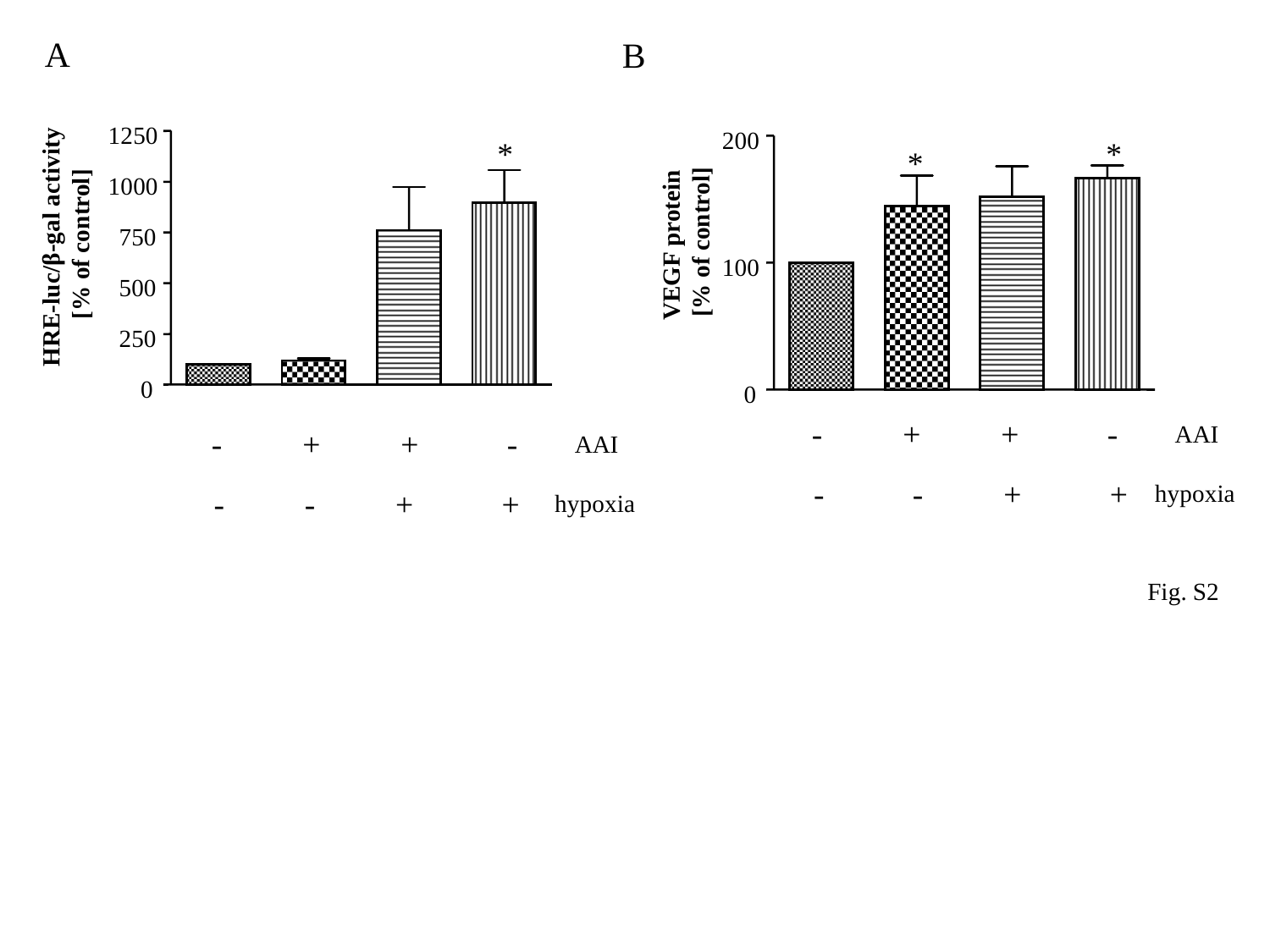

A
B
1250
200
*
*
*
1000
VEGF protein
 [% of control]
HRE-luc/β-gal activity
 [% of control]
750
100
500
250
0
0
 - + + -
 - + + -
AAI
AAI
 - - + +
hypoxia
 - - + +
hypoxia
Fig. S2

## Slide 3
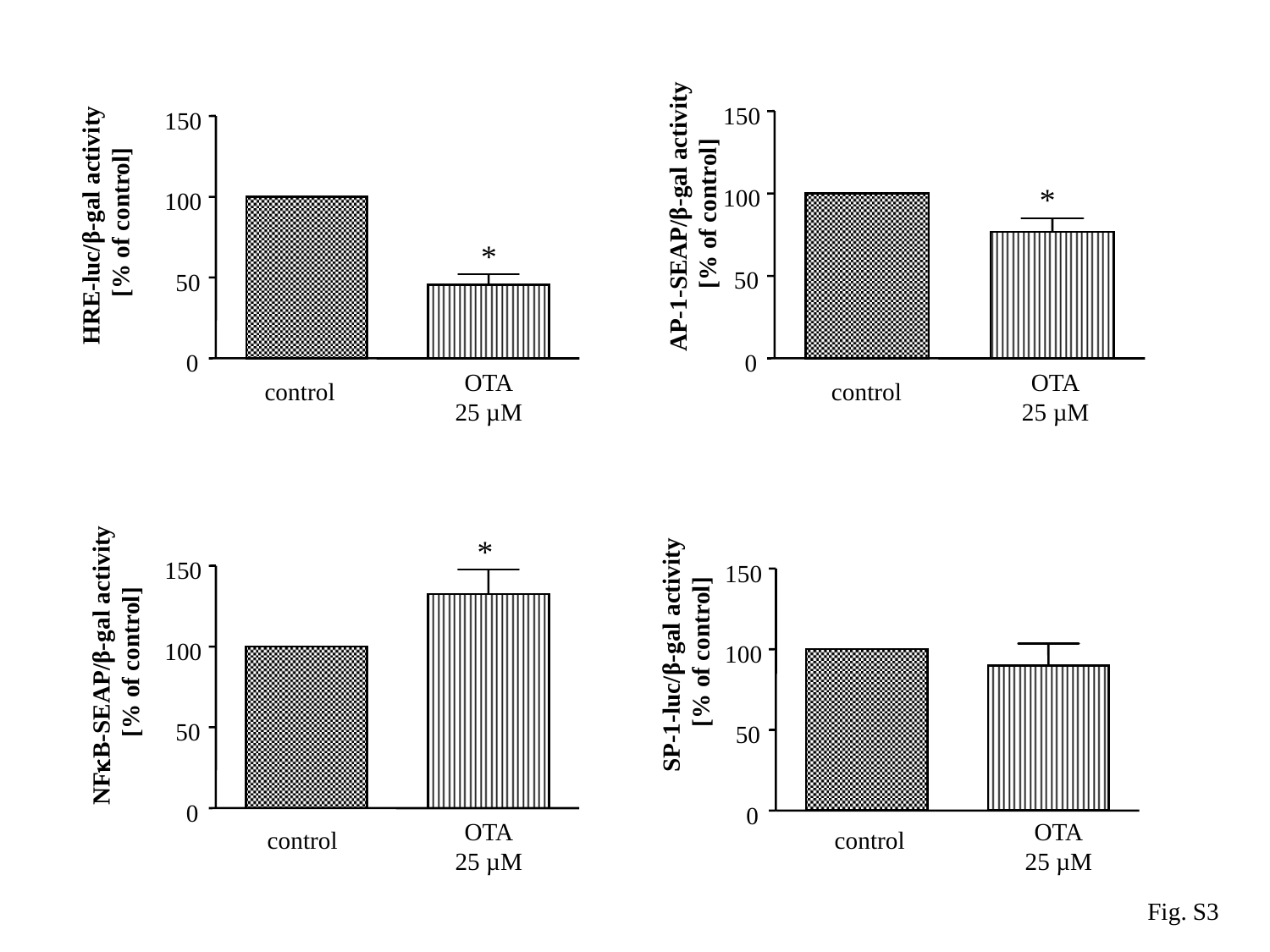

150
150
*
100
100
AP-1-SEAP/β-gal activity
 [% of control]
HRE-luc/β-gal activity
 [% of control]
*
50
50
0
0
OTA
25 µM
OTA
25 µM
control
control
*
150
150
SP-1-luc/β-gal activity
 [% of control]
100
NFκB-SEAP/β-gal activity
 [% of control]
100
50
50
0
0
OTA
25 µM
OTA
25 µM
control
control
Fig. S3
